# A robust modeling framework to investigate transmissible diseases: COVID-19 in Chile as a case study

**DOI:** 10.3389/fpubh.2025.1718363

**Published:** 2026-01-02

**Authors:** Oscar Rojas-Díaz, Patricio Cumsille, Carlos Conca

**Affiliations:** 1 Department of Mathematics and Computer Science, University of Santiago of Chile, Santiago, Chile; 2 Centre for Biotechnology and Bioengineering, University of Chile, Santiago, Chile; 3 Department of Basic Sciences, University of Bío-Bío, Chillán, Chile; 4 Department of Mathematical Engineering and Center for Mathematical Modeling, University of Chile, Santiago, Chile

**Keywords:** epidemiological modeling, parameter optimization, convergence, stability, modeling calibration, COVID-19

## Abstract

**Introduction:**

In the face of the challenges posed by pandemics like COVID-19, the need for accurate mathematical models and methods to predict the impact of outbreaks on public health in real-time is paramount. This research aims to develop a robust, adaptable, and general framework for investigating the dynamics of any transmissible disease. The primary objective of this work is to achieve robust parameter optimization for a general modeling framework, a capability crucial for effectively applying it to reconstruct and predict epidemiological curves for any transmissible disease.

**Methods:**

The main novelty of this work is that our general modeling framework automatically selects a suitable initial parameter vector under general conditions that standard initialization heuristics do not necessarily satisfy, thereby endowing the parameter estimation method with stability and the ability to calibrate any COVID-19 dataset. In addition, we have designed our framework to be adaptable, allowing it to incorporate additional modeling variables, such as ICU admissions beyond infection incidence, and a greater number of time-varying parameters, including the transmission rate, to enhance calibration accuracy. This adaptability ensures that the framework can be tailored to the specific characteristics of different diseases and datasets, enhancing its calibration capabilities.

**Results:**

The main strength of this work lies in the robustness of our general modeling framework. It has demonstrated its ability to optimize modeling parameters that accurately calibrate trends observed in epidemiological curves across all regions of Chile, with varying population sizes and distinct periods/waves of the COVID-19 pandemic, including the effective reproductive number at the national level. In addition, the quantitative measures we calculated further validate the performance of the general modeling framework.

**Conclusion:**

This research represents a significant first step in establishing a robust modeling framework to investigate the dynamics of any transmissible disease. Our findings not only provide a solid foundation for future studies but also have the potential to inform the development of effective disease control strategies, thereby advancing public health.

## 1 Introduction

The present article aims to achieve robust parameter optimization, understood as the ability to accurately calibrate any dataset under general conditions by developing suitable modeling frameworks. To the best of our knowledge, this crucial aspect has not been addressed in the literature, and it is key to effectively reconstructing and predicting epidemiological curves for any transmissible disease. Given the limitations in managing a pandemic like COVID-19 (1), this aspect is desirable for forecasting an outbreak's impact on the public health system in real time, providing scientific evidence to help inform policymakers' decisions.

Our primary objective is to endow a previously developed general modeling framework with the strength described above. Our framework employs a hybrid approach that combines mathematical modeling with a practical identifiability approach (2). The first involves suitable compartmental epidemiological modeling, whereas the second relies primarily on parameter optimization for model calibration. We conceived it by generalizing and refining previous modeling and numerical methods introduced by Cumsille et al. (3). We applied it to accurately track and predict the number of patients hospitalized in the intensive care unit (ICU) and to assess COVID-19 vaccination in Chile (4).

The main novelty of our work lies in parameter initialization that satisfies general conditions solely dependent on the parameter space, which is tied to the epidemiology of the studied transmissible disease. As a result, our framework is robust since it can successfully calibrate any dataset and reproduce the national effective reproduction number, as substantiated by fitting COVID-19 incidence curves across all of Chile's regions, with varied mobility levels, geographic locations, and population sizes during the pandemic. We remark that this ability is not trivial and, indeed, we required intensive numerical experiments to achieve it since it has no direct or unique implementation. The main difficulty is that parameter identification is an ill-posed inverse problem, as its solution lacks stability and uniqueness with respect to parameter values (5). In particular, modeling errors and measurement noise can be amplified considerably (6), leading to numerical instability, yielding poor data calibration. It is worth noting that, in the current work, the leading cause of instability lies in the evaluation of the modeling solution rather than in the optimization of the objective function. Thus, we focus on providing stability to the model solver, which, in turn, implies stability for the optimization solver, thereby avoiding the need to resort to, e.g., Tikhonov regularization.

The literature on COVID-19 modeling is vast. Thus, we only examine a few papers that are important for their applications and similarities with the present work. For example, Zhenzhen et al. (7) studied a model with “long memory” to describe the multi-wave peaks in the COVID-19 dynamics, where “long memory” enables the prediction of peaks using nonlocal terms, allowing one to include an arbitrary long history of the disease. Indeed, the authors obtain a model with time delays for a particular nonlocal term. Also, the authors modeled vaccination as an impulsive term that reduces susceptibility.

Moreover, Ghosh et al. (8) derived a model with a time delay, where the last term represents the disease duration, i.e., the average time it takes for infected individuals to recover or die. Also, (9) investigated a SEIR-type model with time delay and vaccination control. Also, they simulated vaccination as a control that decreases susceptibility, similar to the generalization we use in the present work. Finally, our current work builds upon the model developed by (3), which shares common elements with some of the works cited here. Indeed, in the cited reference, we introduce the same time delay that models the average time to recover or die. In addition, we could interpret our previous model as incorporating a long-memory effect, in the sense that it allows reproducing multi-wave peaks depending on parameter values, as we showed.

Finally, according to Moore et al. (10), vaccination protects in four ways: against infection, against symptoms, against severe disease, and against onward transmission. However, even accounting for some of the vaccine's protective mechanisms, the model can become very complex, making it difficult to estimate parameters reliably. Therefore, our present work maintains model simplicity, which is critical for a practical identifiability approach. Even so, it remains a challenge to do so in real time, as required to anticipate epidemiological scenarios and predict hospital load on the healthcare system, as was the case during the first year of the pandemic (before vaccination). In this regard, we assume that vaccination protects against infection by diminishing susceptibility, as reported in the cited literature, which entails adding several meaningful parameters associated with vaccination.

We organize this article as follows. Section 2 describes the general modeling framework, comprising a robust parameter optimization. Section 3 describes the errors, robustness measures, and the effective reproduction number. In Section 4, we present results from the general modeling framework on its performance, modeling evaluation stability, adaptability, and robustness, i.e., its calibration accuracy under general conditions. In Section 5, we analyze the results to demonstrate that our general modeling framework is stable, adaptable, and robust. Finally, in Section 6, we present conclusions and outline future directions.

## 2 General modeling framework

This section provides a summary of the general modeling framework, which comprises the general epidemiological modeling described in Section 2.1 and a practical identifiability approach that enables modeling parameter calibration for data of any transmissible disease, as explained in Section 2.2. Finally, in Section 2.3, we present the *general percentage decrease technique*, a component of the practical identifiability approach, that enables robust parameter optimization.

### 2.1 General epidemiological modeling

In previous works (3, 4), we developed epidemiological models containing long-memory terms that yield accurate multi-wave reproduction peaks for suitable parameter values. General epidemiological modeling developed by Cumsille et al. (4) partly consists of the following nonlinear delayed differential equations (DDE) system:


$$\begin{array}{l} \frac{dS}{dt}\left(t\right)=-\frac{\beta \left(t\right)}{N_{pop}}S\left(t\right)I\left(t-\tau_{1}\right)-\alpha \left(t\right)S\left(t-\tau_{3}\right)+\delta \left(t\right)S\left(t-\tau_{4}\right), \end{array}$$ (1a)

$$\begin{array}{l} \frac{dI}{dt}\left(t\right)=\frac{\beta \left(t\right)}{N_{pop}}S\left(t\right)I\left(t-\tau_{1}\right)-\gamma \left(t\right)I\left(t-\tau_{2}\right)-\eta \left(t\right)I\left(t-\tau_{5}\right), \end{array}$$ (1b)

$$\begin{array}{l} \frac{dR}{dt}\left(t\right)=\gamma \left(t\right)I\left(t-\tau_{2}\right)+\alpha \left(t\right)S\left(t-\tau_{3}\right)-\delta \left(t\right)S\left(t-\tau_{4}\right) \end{array}$$ (1c)

$$\begin{array}{c} +\kappa \left(t\right)U\left(t-\tau_{6}\right),\\ \frac{dU}{dt}\left(t\right)=\eta \left(t\right)I\left(t-\tau_{5}\right)-\kappa \left(t\right)U\left(t-\tau_{6}\right). \end{array}$$ (1d)

The variables of the nonlinear DDE system given by Equation 1 are *S*, *I*, and *R*, which describe the susceptible, infected, and recovered individuals, and *U*, which accounts for the sum of the patients hospitalized in the ICU plus the dead confirmed by the transmissible disease, respectively. We consider a piecewise reconstruction of time-varying parameters α(*t*), β(*t*), γ(*t*), δ(*t*), η(*t*), and κ(*t*) (see Table 1). General epidemiological modeling has *N*_θ_: = 6*N*_θ_0__+9 parameters collected in the vector $\theta \in {\mathbb{R}}^{N_{\theta }}$ defined by Equation 2, where *N*_θ_0__ is the number of time subintervals equally spaced to interpolate parameters ***θ***_**0**_, defined by Equation 2a, which reconstruct transmission rate and transition rates previously mentioned. In modeling calibration, we used *N*_θ_0__ = 20 time subintervals to describe the transmission rate and transition rates; thus, we estimated *N*_θ_ = 129 parameters for each dataset, which presented a computational challenge, as we had to develop efficient programming, which we made accessible to the scientific community in (11). It is worth noting that this high number of parameters applies only to ***θ***_**0**_ since the rates are calculated by pieces to map the entire pandemic, accounting for temporal variability across different periods/waves. In ***θ***_**1**_ defined by Equation 2b, parameters *a* and *k* describe the minimum and the steepness parameter of *f*(*t*) given by Equation 3, and *I*_*thr*_ is the threshold of detected infections such that Equation 4 holds. Parameter *f*(*t*) is the fraction of detected and fitted infections by modeling since the reported infections *I*_*r*_(*t*) are underestimated (3). Thus, *I*_*f*_(*t*) = *f*(*t*)*I*(*t*) are the new cases of modeled infections detected in day *t*. **τ** are constant time delays that represent meaningful epidemiological parameters that vary in the ranges provided in Table 1, which for COVID-19 were obtained from the references (4, 12). The initial values for vector **τ** are $\tau_{1}^{\left(0\right)}=5,\tau_{2}^{\left(0\right)}=14$, which correspond to the mean for τ_*i*_ for *i* = 1, 2 in the COVID-19 case (13), while the other initial values are the midpoint of the ranges in Table 1. The details of the model given by equations in System 1, *I*_*f*_(*t*) = *f*(*t*)*I*(*t*), and Equation 3, and parameter optimization are described in depth by Cumsille et al. (4). Table 1 defines the parameters of general epidemiological modeling, collected in the vector **θ** as given by the equations in System 2.


$$\theta_{0}={\left[\alpha ,\;\beta ,\;\gamma ,\;\delta ,\;\eta ,\;\kappa \right]}^{T}\in {\mathbb{R}}^{6N_{\theta_{0}}},$$ (2a)

$$\theta_{1}={\left[a,\;k,\;I_{thr}\right]}^{T}\in {\mathbb{R}}^{3},$$ (2b)

$$\tau ={\left[\tau_{1},\;\tau_{2},\;\tau_{3},\;\tau_{4},\;\tau_{5},\;\tau_{6}\right]}^{T}\in {\mathbb{R}}^{6},$$ (2c)

$$\theta ={\left[\theta_{0},\;\theta_{1},\;\tau \right]}^{T}\in {\mathbb{R}}^{N_{\theta }},\;N_{\theta }=6N_{\theta_{0}}+9.$$ (2d)

$$\begin{array}{l} f\left(t\right)=1+\frac{a-1}{1+e^{-k\left(I\left(t\right)-I_{thr}\right)}}. \end{array}$$ (3)

$$I(t)\ll I_{thr}\Rightarrow f(t)\approx 1,$$ (4a)

$$I(t)\gg I_{thr}\Rightarrow f(t)\approx a.$$ (4b)

**Table 1:** Parameters of general epidemiological modeling.

**Symbol**	**Description**	**Unit**	**Range**
τ_1_	Mean incubation time	days	1–14
τ_2_	Mean time to recover for mild cases	days	1–21
τ_3_	Mean time from susceptible to recovery (by vaccination immunity)	days	1–14
τ_4_	Mean duration of immunity	days	1–240
τ_5_	Mean time from infected to ICU	days	14–56
τ_6_	Mean time from ICU to recover	days	21–42
***α***	Mean transition rate from susceptible to recovered (immunity rate)	days^−1^	
***β***	Mean transmission rate	days^−1^	
***γ***	Mean recovery rate for mild cases	days^−1^	
***δ***	Mean transition rate from recovered to susceptible (immunity decay rate)	days^−1^	
***η***	Mean transition rate from infected to ICU	days^−1^	
***κ***	Mean recovery rate for ICU patients	days^−1^	
*a*	Minimum fraction of detected infections	–	
*k*	Steepness parameter of the fraction of detected infections	inhabitants^−1^	
*I*_*thr*_	Threshold of detected infections	inhabitants	

### 2.2 Practical identifiability approach

To carry out the practical identifiability approach, which relies primarily on parameter optimization for model calibration, we must solve the following parameter estimation problem: Given a (processed) dataset accounting for a specific transmissible disease dynamics, compute a parameter vector ***θ*** such that general epidemiological modeling fits data by least-squares. The dataset is the time observations $\{[I_{r}(t_{j}),\;U_{r}(t_{j})]\;:\;j=1,\cdots ,N^{i}\}\text{ for every }i=1,\ldots ,N,$ where $I_{r}^{i}\left(t_{j}\right)=I_{r}\left(t_{j}\right)$ are the observations of variable *I*_*f*_(*t*_*j*_), corresponding to the case incidence confirmed by RT-PCR tests for the *i*-dataset in day *t*_*j*_, and $U_{r}^{i}\left(t_{j}\right)=U_{r}\left(t_{j}\right)$ are the observations of variable *U*(*t*_*j*_), corresponding to the patients hospitalized in the ICU plus the dead confirmed by the disease for the *i*-dataset in day *t*_*j*_, *N*^*i*^ is the *i*-dataset size, and *N* is the total number of studied datasets. In this work, we study *N* = 16 datasets that cover the entire Chilean territory. We remark that, throughout this article, we omit the dependence on *i* for simplicity. To solve the parameter estimation problem, we must estimate a vector $\theta \in {\mathbb{R}}^{N_{\theta }}$, given by System 2, minimizing the cost function (Equation 5).


$$\begin{array}{l} SS\left(\theta \right)=\overset{N^{i}}{\underset{j=1}{\sum }}\left[{\left(\frac{I_{r}\left(t_{j}\right)-I_{f}\left(t_{j},\theta \right)}{I_{f}\left(t_{j},\theta \right)+\varepsilon }\right)}^{2}+{\left(\frac{U_{r}\left(t_{j}\right)-U\left(t_{j},\theta \right)}{U\left(t_{j},\theta \right)+\varepsilon }\right)}^{2}\right] \end{array}$$ (5)

The cost function defined in Equation 5 corresponds to the sum of squares of the differences between the data and the model relative to the model. Variables *I*_*f*_ and *U* are the solution of general epidemiological modeling given by equations in System 1, *I*_*f*_(*t*) = *f*(*t*)*I*(*t*), and Equation 3 at (*t*_*j*_, ***θ***) for *j* = 1, ⋯ , *N*^*i*^, for a particular parameter vector $\theta ={\left[\theta_{0},\;\theta_{1},\;\tau \right]}^{T}\in {\mathbb{R}}^{N_{\theta }}$ given by equations in System 2. In Equation 5, we introduce a parameter 0 ≤ ε ≤ *tol* to avoid division by zero and chosen as ε = 0 when *I*_*f*_(*t*_*j*_, **θ**)>0 and *U*(*t*_*j*_, **θ**) for all *j*, where *tol* = 1.0*e*−6. Another alternative to stabilize the computation of relative residuals is to add 1 to each datapoint in the target dataset, ensuring that the numerical modeling solution is always greater than 0, thereby allowing us to set ε = 0 and obtain a calibration as good as that for the original dataset. To optimize *SS*(**θ**) (Equation 5) for the *i*-dataset, for all *i* = 1, …, *N*, we apply the *lsqnonlin* MATLAB solver that minimizes the sum of squares by the well-known *Trust-Region-Interior-Reflective (TIR)* Algorithm (14).

The modeling parameters **θ**_1_ given by Equation 2b depend on each dataset. As we are searching for an overall numerical method that makes robust parameter optimization for every dataset under general conditions, one can initialize **θ**_1_ strategically by defining *I*_*thr*_ between the minimum and maximum of the reported case incidence *I*_*r*_ computed among all studied datasets (4). We define the modeling parameters **τ** given by Equation 2c within reasonable bounds from an epidemiological viewpoint. Used vector τ values are given in Table 1 according to references (4, 12) for COVID-19.

### 2.3 General percentage decrease technique

Based on epidemiological considerations and our numerical experience, we generalized and improved the percentage decrease technique to select initial modeling parameters as a fraction of the upper bounds of the parameter space, endowing the practical identifiability approach with robust parameter optimization. To the best of our knowledge, this crucial aspect has not been addressed in the literature, and it is key to extending our general modeling framework to effectively reconstruct and predict epidemiological curves for any transmissible disease. The extension of our technique is implemented in Algorithm 1 for initial parameter selection, which is crucial for optimizing the cost function *SS*(**θ**) defined by Equation 5 suitably. The technique focuses on initializing modeling parameters **θ**_0_ as a fraction of the upper bound UB_**θ**_0__ for the parameter vector **θ**_0_ as a guide for computation. In processing each dataset, the fraction value is iterated until Algorithm 1 meets the following stopping conditions: (i) the computed cumulative incidence *I*_*f*_(*t*_*j*_, **θ**) is less than the observed cumulative incidence *I*_*r*_(*t*_*j*_) over *t*_*j*_ for *j* = 1, …, *N*^*i*^, and (ii) the computed incidence is greater or equal to 0 for all *t*_*j*_, *j* = 1, …, *N*^*i*^. Mathematically, we can formulate conditions (i) and (ii) by Equations 6a and 6b, respectively.


$$\begin{array}{l} \overset{N^{i}}{\underset{j=1}{\sum }}I_{r}\left(t_{j}\right)>\overset{N^{i}}{\underset{j=1}{\sum }}I_{f}\left(t_{j},\theta \right) \end{array}$$ (6a)

$$\begin{array}{l} \forall t_{j},j=1,\ldots ,N^{i},I_{f}\left(t_{j},\theta \right)\geq 0. \end{array}$$ (6b)

We remark that condition (Equation 6a) means that the area under the observed curve (*t*_*j*_, *I*_*r*_(*t*_*j*_)) is greater than the area under the modeled curve (*t*_*j*_, *I*_*f*_(*t*_*j*_, **θ**)) since both sums in Equation 6a are approximations for the integral over the calibration period taking Δ*t* = 1 day. The iterative procedure we devised to determine the fraction values (Algorithm 1) effectively works independently of each dataset, as confirmed by extensive numerical experiments. This study required intensive numerical simulations, as our approach is complex and lacks a direct, unique implementation. The main difficulty is that parameter identification is an ill-posed inverse problem, as its solution lacks specific stability properties, leading to non-uniqueness in parameter optimization (5). In particular, modeling errors and measurement noise can be amplified considerably (6), possibly leading to numerical instability and divergence of the optimization solver that, in our numerical experience, occurs when implementing a careless parameter optimization technique. Algorithm 1 is a controlled-parameter initialization technique that reinitializes only ***θ***_**0**_ to avoid instabilities in the dde23 MATLAB solver (15) when evaluating numerical solutions to the DDE system (System 1). In contrast, other initialization heuristics, such as random ones, may produce instabilities in the dde23 solver, which, in our numerical experience, are mainly due to the solver's sensitivity to changes in derivatives, especially near critical points.

Algorithm 1 General percentage decrease technique. 
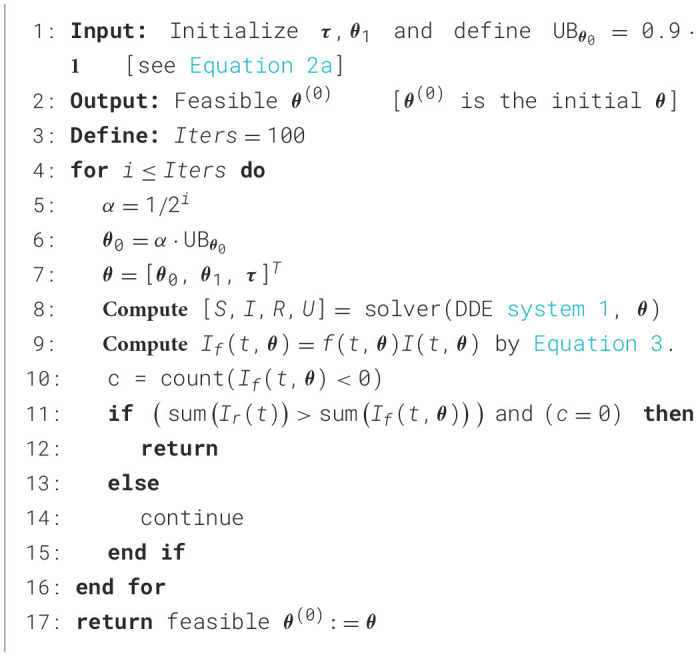

## 3 Methods

**Figure 1:**
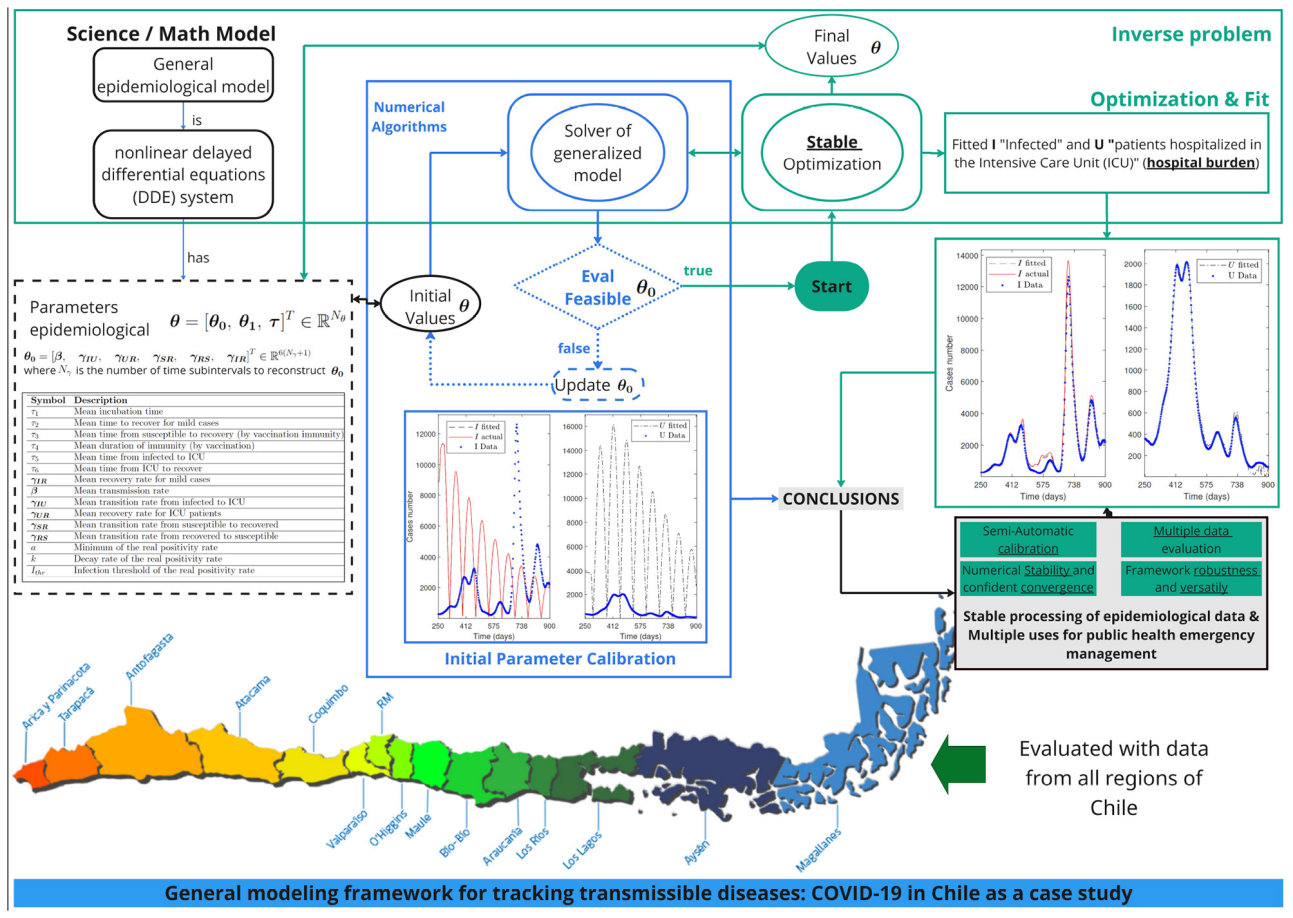
Graphical abstract. The plot depicts a conceptual scheme, the main results, and the conclusions of the general modeling framework. It consists of general epidemiological modeling (GEM) and robust parameter optimization with suitable initial parameter selection. We applied our framework to calibrate COVID-19 datasets across all regions of Chile. It consistently calibrates case incidence for infections and ICU admissions, enabling assessment of the hospital burden on the public health system. In conclusion, our framework is adaptable, robust, and stable, enabling the stable processing of epidemiological data for multiple uses in public health emergency management.

In this section, we describe the methods to demonstrate the stability, adaptability, and robustness of our general modeling framework. Figure 1 presents a conceptual scheme for robust parameter optimization that incorporates the general percentage decrease technique (Algorithm 1) for parameter initialization.

### 3.1 Measurement of the error and robustness of the optimization

To demonstrate the performance of the general modeling framework, we present our results by plotting the relative error distributions of the general epidemiological modeling calculation for various modeling variables and their corresponding observed data. We calculate the *errors relative to data* defined by Equation 7:


$$\begin{array}{l} {\text{RE}}_{X}\left(t_{j},\theta \right)=\frac{X_{r}\left(t_{j}\right)-X\left(t_{j},\theta \right)}{X_{r}\left(t_{j}\right)+\varepsilon }\;j=1,\ldots ,N^{i}, \end{array}$$ (7)

where *X* stands for every modeling variable, *X* = *S*,  *I*_*f*_,  *R*,  *U*, and *X*_*r*_ denotes the corresponding observed data, *X*_*r*_ = *S*_*r*_,  *I*_*r*_,  *R*_*r*_,  *U*_*r*_. Again, a small parameter ε>0 is chosen to avoid division by zero if required, which is also valid for the RMSE defined by Equation 9. In addition, we will show in tables, which display quantitative metrics of performance, the mean relative error defined as the average of the absolute value of the relative errors in the respective time interval defined by Equation 8:


$$\begin{array}{l} {\text{MRE}}_{X}\left(\theta \right)=\frac{1}{N^{i}}\overset{N^{i}}{\underset{j=1}{\sum }}|{\text{RE}}_{X}\left(t_{j},\theta \right)|, \end{array}$$ (8)

where RE_*X*_(*t*_*j*_, **θ**) is defined in Equation 7.

We also plot the case incidence fitted *I*_*f*_, calculated *I*, and reported *I*_*r*_ (denoted as “*I* fitted”, “*I* calculated”, and “*I* reported” in legends at left panels of figures). In addition, we plot the fitted variable *U* and the reported *U*_*r*_, which represent the sum of patients in the ICU and those confirmed dead by COVID-19 (denoted as “U fitted” and “U reported” in the legends of the right panels of the figures).

The specific robustness metrics that we display are the sum of squares' approximate optimal value $\text{SS}\left(\hat{\theta }\right)$ evaluated in the numerical approximation to a suitable minimum, $\hat{\theta }$, the root mean squared error (RMSE) for *I*_*f*_ and *U* defined by Equation 9 for *X* = *I*_*f*_ and *X* = *U*, the norm of step $\hat{\delta }$ defined as the distance between $\hat{\theta }$ and the parameter vector computed in the previous iteration, the mean relative error for *I*_*f*_ and *U*, ${\text{MRE}}_{I_{f}}\left(\hat{\theta }\right)$ and ${\text{MRE}}_{U}\left(\hat{\theta }\right)$, defined by Equation 8, the calibration period, and the geographic location for every dataset; see Figure 1 for reference.


$$\begin{array}{l} {\text{RMSE}}_{X}\left(\theta \right)={\left[\frac{1}{N^{i}-N_{\theta }}\overset{N^{i}}{\underset{j=1}{\sum }}{\left(\frac{X_{r}\left(t_{j}\right)-X\left(t_{j},\theta \right)}{X\left(t_{j},\theta \right)+\varepsilon }\right)}^{2}\right]}^{1/2}. \end{array}$$ (9)

### 3.2 Estimation of the effective reproduction number

In addition to demonstrating calibration performance for COVID-19 datasets of all regions of Chile, we compute and plot the effective reproduction number *R*_*e*_(*t*) using the incidence curve at a national level.

From Equation 1b, we note that *I*′(*t*)>0 if and only if


$$\begin{array}{l} R_{e}\left(t\right):=\frac{\beta \left(t\right)S\left(t\right)I\left(t-\tau_{1}\right)}{N_{pop}\left[\gamma \left(t\right)I\left(t-\tau_{2}\right)+\eta \left(t\right)I\left(t-\tau_{5}\right)\right]}>1 \end{array}$$

Also, factorizing Equation 1b by γ(*t*)*I*(*t*−τ_2_)+η(*t*)*I*(*t*−τ_5_), we arrive at the “calculated” effective reproductive number:


$$\begin{array}{l} R_{e}\left(t\right)=\frac{I^{\prime }\left(t\right)}{\gamma \left(t\right)I\left(t-\tau_{2}\right)+\eta \left(t\right)I\left(t-\tau_{5}\right)}+1. \end{array}$$ (10)

In addition, we compute the “fitted” effective reproductive number:


$$\begin{array}{l} {\left(R_{e}\right)}_{f}\left(t\right):=\frac{f\left(t\right)I^{\prime }\left(t\right)}{\gamma \left(t\right)f\left(t-\tau_{2}\right)I\left(t-\tau_{2}\right)+\eta \left(t\right)f\left(t-\tau_{5}\right)I\left(t-\tau_{5}\right)}+1. \end{array}$$ (11)

We note that the analogy between *R*_*e*_(*t*) and (_*R*_*e*_)*f*_(*t*) is the same as for *I*(*t*) and *I*_*f*_(*t*) = *f*(*t*)*I*(*t*), where *I*_*f*_(*t*) is the “fitted” case incidence, whereas *I*(*t*) is the “calculated” case incidence, which is unknown since it models the reported and unreported incidence case.

In our numerical calculation of *R*_*e*_(*t*), we preferred formula 10 for stability, where we approximate the derivative *I*′(*t*) by the classical second-order centered finite-difference formula. In our numerical results, we plot the “modeled” effective reproductive number *R*_*e*_(*t*) calculated by Equation 10, the “fitted” effective reproductive number (_*R*_*e*_)*f*_(*t*) calculated by Equation 11, and the “observed” effective reproductive number calculated by using the reported case incidence *I*_*r*_(*t*) by Equation 12:


$$\begin{array}{l} {\left(R_{e}\right)}_{ob}\left(t\right):=\frac{I_{r}^{\prime }\left(t\right)}{\frac{1}{14}I_{r}\left(t-14\right)+\frac{1}{35}I_{r}\left(t-35\right)}+1, \end{array}$$ (12)

assuming that the average time to recover for mild cases is 14 days (4), and to be admitted to the ICU due to COVID-19 is 35 days (the midpoint of the range for τ_5_; see Table 1), respectively.

### 3.3 Data, hardware, and software

The datasets used in this research are available in (11) and correspond to the observed case, [*I*_*r*_(*t*_*j*_),  *U*_*r*_(*t*_*j*_)], for all regions of Chile, geographic locations (North, Center, and South), population sizes, and distinct periods/waves of the COVID-19 pandemic. Before parameter initialization and optimization for modeling calibration, we processed the datasets by cleaning, smoothing, and interpolation (4). We selected different periods of the COVID-19 pandemic to test the robustness of our general modeling framework, focusing on its ability to calibrate the entire pandemic and its various waves. The precise periods in days for the selected datasets are 30–950, 100–900, or 100–950 (almost the whole pandemic), 30–250 (first wave), 250–950 (all the pandemic except for the first wave), 250–600 (good part of the pandemic except for the first wave). We do not include graphs for all experiments to avoid overextending the presentation. We note that day 1 corresponds to March 3, 2020, the date the COVID-19 pandemic began, when the Health Ministry reported the first confirmed case in Chile.

The hardware used is a data science workstation equipped with an Intel Core i9-14900K processor, 32 logical cores, and 128 GB of RAM. The software used for development and experimentation is MATLAB R2024a©. The options we chose for the lsqnonlin solver are function tolerance 1.0*e* − 12, the maximum number of function evaluations 40, 000, optimality tolerance 1.0*e*−12, and step tolerance 1.0*e*−10. We also used the parallel computing MATLAB toolbox to leverage the workstations' multi-core processors.

## 4 Results

This section presents the results obtained from the general modeling framework. We will demonstrate that our framework, which sheds light on the understanding of epidemiological curves (4), can calibrate datasets corresponding to the entire territory of Chile. Figure 1 summarizes the main results and conclusions obtained by applying the general modeling framework for all datasets corresponding to COVID-19 cases observed across all regions of Chile, with varying mobility levels, geographic locations, and population sizes, during the pandemic's duration.

This section is organized as follows. First, we quantify the performance of the general percentage-decrease technique and determine whether it endows the practical identifiability approach with numerical stability. Second, we optimize different objective functions to determine whether our general modeling framework can better calibrate the trend of the observed epidemiological curves by incorporating additional modeling variables and parameters into the parameter estimation problem. Finally, we quantify the performance of the general modeling framework to demonstrate that it can robustly optimize parameters under general conditions ensured by the general percentage decrease technique.

### 4.1 General modeling framework numerical stability

Algorithm 1 selects suitable initial modeling parameters for stable numerical parameter optimization, as demonstrated below. In effect, to test stability, we used COVID-19 case incidence in the Metropolitan Region (RM in Figure 1). Table 2 quantifies the computational cost and the mean relative error (MRE) as defined in Equation 8 over the period from 250 to 900 days (excluding the first wave), calculated using the general percentage decrease technique. Note that we are not calibrating general epidemiological modeling, but rather testing the stability of Algorithm 1 before parameter optimization.

**Table 2:** General percentage decrease technique results.

**Computational cost**			
**Cost**	**Solver dde23 (with NaNs)**	**Solver dde23 (without NaNs)**	**Total**
Mean time	1.5 sec	0.05 sec	6.1 sec
Mean latency	1.0 sec	0.1 sec	4.2 sec
Iters	4	3	7
**Mean relative error**			
Iters 1-4	NaNs	-	-
Iters 5-7 (*I*)	-	[5.36*e*+00, 1.94*e*+00, 1.26*e*+00]	-
Iters 5-7 (*R*)	-	[5.97*e*+00, 2.11*e*+00, 1.35*e*+00]	-
Iters 5-7 (*U*)	-	[1.81*e*+01, **1.34e+00**, **5.54e-01**]	-

In terms of computational cost, Table 2 shows the mean time, latency^1^, and iterations that take Algorithm 1 to meet the stopping criteria described by conditions (i) and (ii) given by Equations 6 for the 751 datapoints in the period 250-900 days for RM.

**Figure 2:**
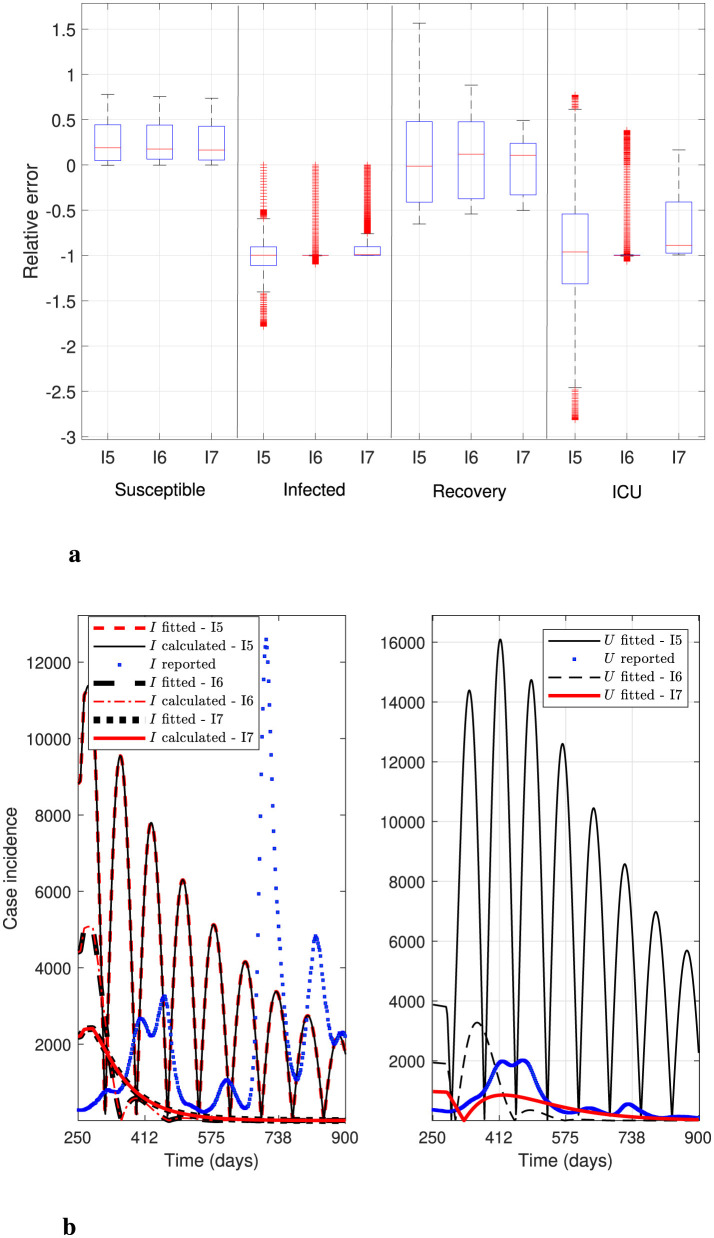
General percentage decrease technique results: iterations 5–7. Results correspond to the daily case incidence evaluated in the parameters obtained by running parameter initialization Algorithm 1. **(a)** shows the relative error distribution for all modeling variables, and **(b)** depicts incidence curves for variables *I*_*f*_, *I*, and *U* in the RM of Chile. Algorithm 1 determines the initial parameter vector at iteration 7, when the *I*, *I*_*f*_, and *U* curves stabilize, enabling robust parameter optimization.

Figure 2 below shows the general percentage decrease technique results in iterations 5, 6, and 7 (marked as I5, I6, and I7 in Figure 2). Specifically, it plots the curves generated by Algorithm 1 to test stability before applying the optimization solver. It depicts box-and-whisker plots of relative error distributions for the different modeling variables and their corresponding data, as defined by Equation 7 (top panel). It also depicts the case incidence *I*_*f*_ and *I* evaluated at ***θ***^**(0)**^ in iterations 5-7, and the reported case incidence *I*_*r*_ (denoted as “*I*_*f*_ fitted”, “*I* calculated”, and “*I* reported” in the legend of the bottom-left panel of the figure). In addition, it plots the sum of patients in the ICU plus those confirmed dead by COVID-19, i.e., the variable *U*, calculated at ***θ***^**(0)**^ in iterations 5-7, and *U*_*r*_ reported (denoted as “*U* fitted” and “*U* reported” in the legend in the bottom-right panel of the figure).

### 4.2 General modeling framework adaptability

**Figure 3:**
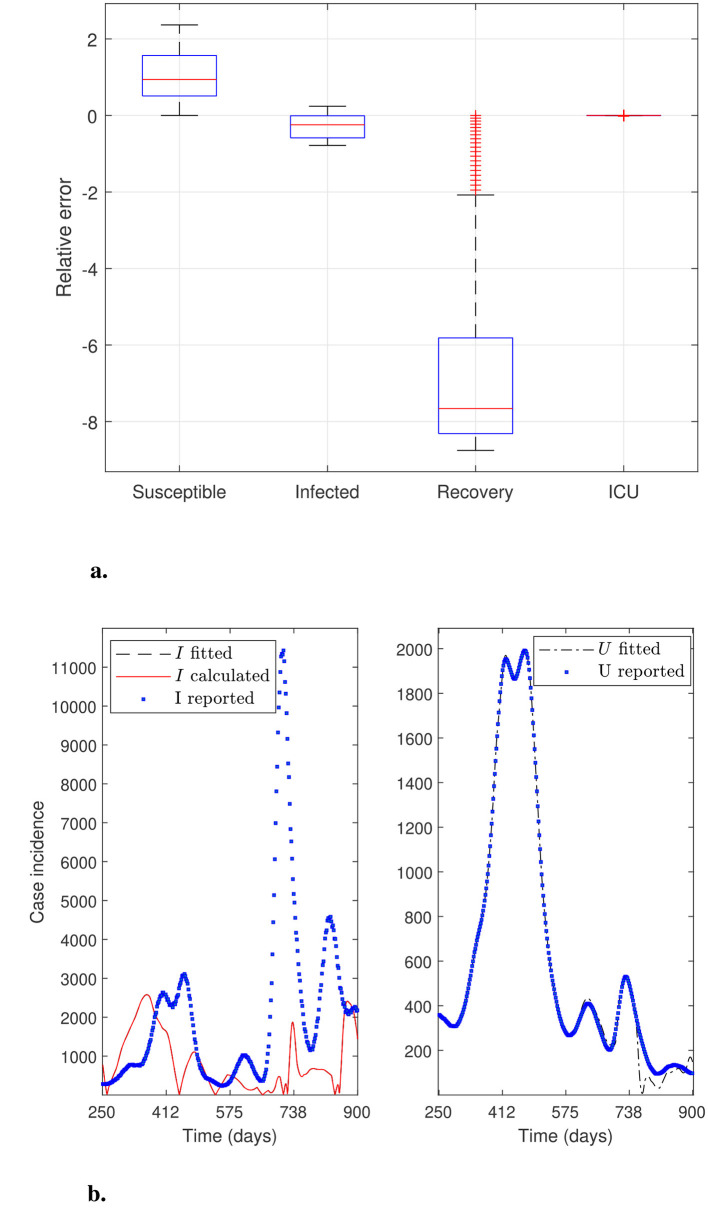
General modeling framework calibration using only variable *U*. Results correspond to the daily case incidence fitted by minimizing the objective function defined by Equation 5, keeping only the relative residuals for variable *U*. **(a)** depicts the relative error distribution of all modeling variables, and **(b)** incidence curves in the RM of Chile for variables *I*, *I*_*f*_, and *U*. Calibration for the variables *I* and *I*_*f*_ (not fitted) is poor, whereas that for the fitted variable *U* is outstanding.

**Figure 4:**
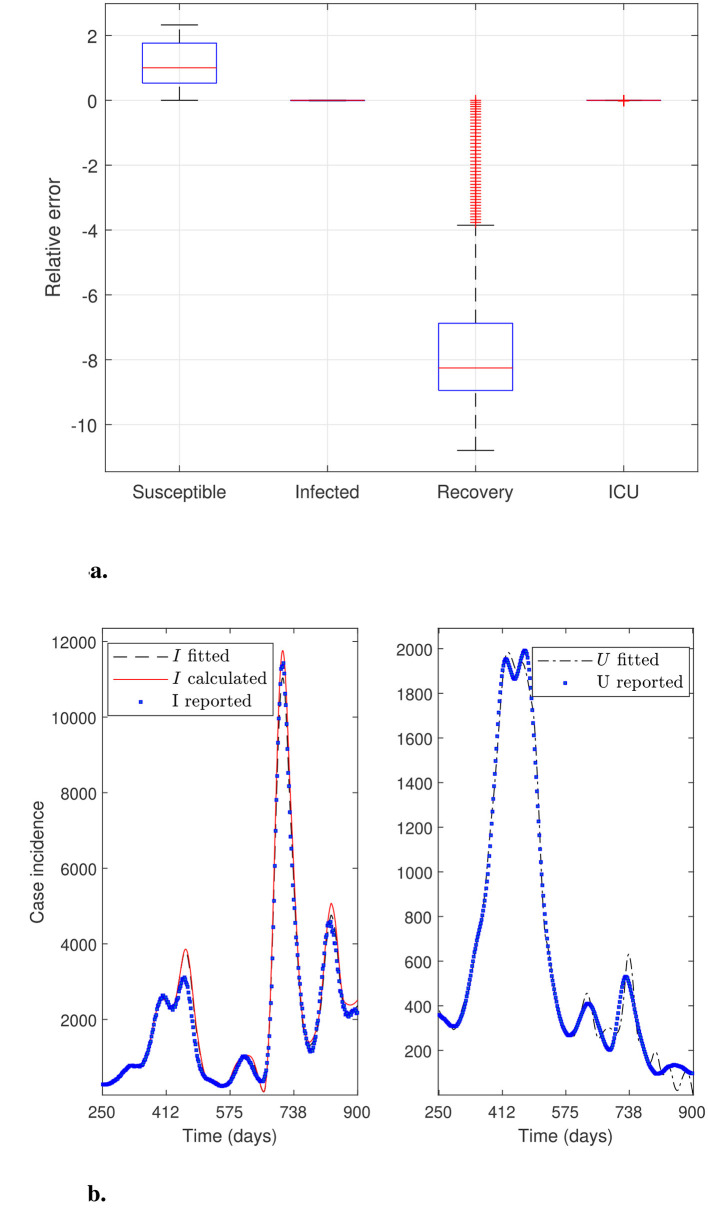
General modeling framework calibration using variables *I*_*f*_ and *U*. Results correspond to the daily case incidence fitted by minimizing the objective function defined by Equation 5. **(a)** depicts the relative error distribution of all modeling variables, and **(b)** incidence curves in the RM of Chile for variables *I*, *I*_*f*_, and *U*. Calibration for fitted variables *I*_*f*_ and *U* is relatively good, except for a spurious peak around day 500 for *I*_*f*_ and *I*, and little oscillations for variable *U* near the end of the third wave.

**Figure 5:**
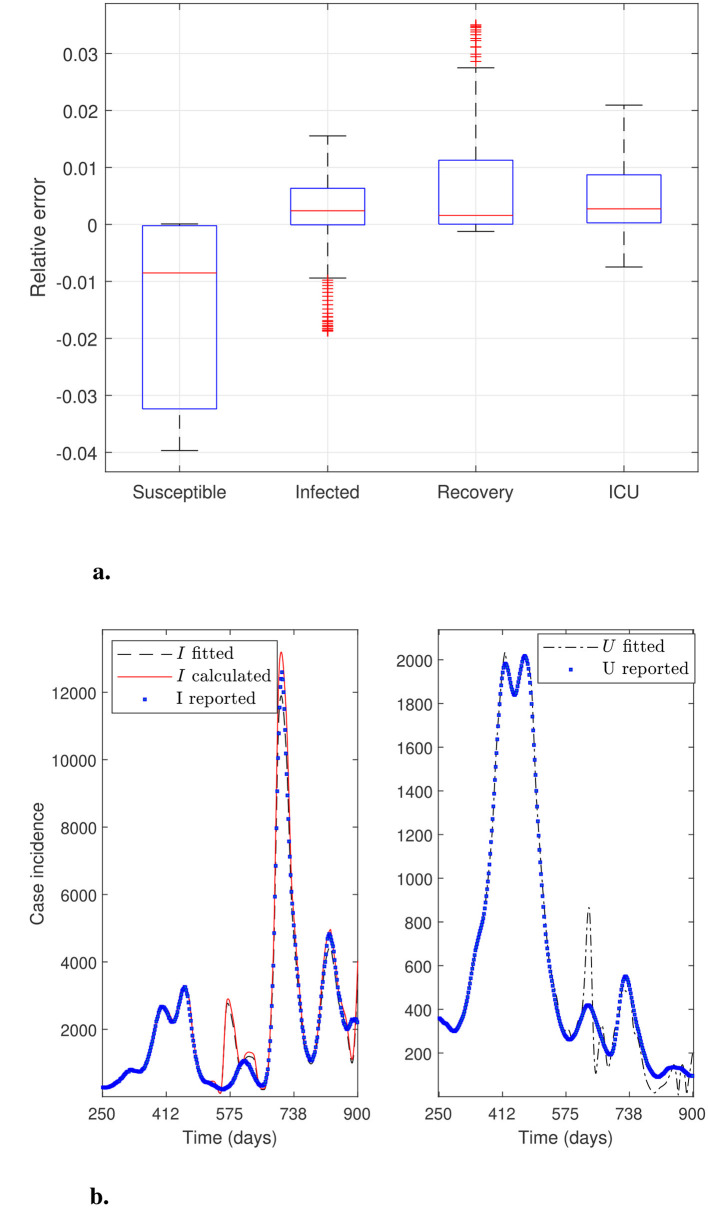
General modeling framework calibration using variables *I*_*f*_, *R*, and *U*. Results correspond to the daily case incidence fitted by minimizing the objective function defined by Equation 5, adding the relative residuals for variable *R*. **(a)** depicts the relative error distribution of all modeling variables, and **(b)** incidence curves in the RM of Chile for variables *I*, *I*_*f*_, and *U*. Calibration is relatively good, except for a spurious peak for *I*_*f*_ and *I* around day 575, and a few oscillations for the fitted variable *U* near the end of the third wave.

**Figure 6:**
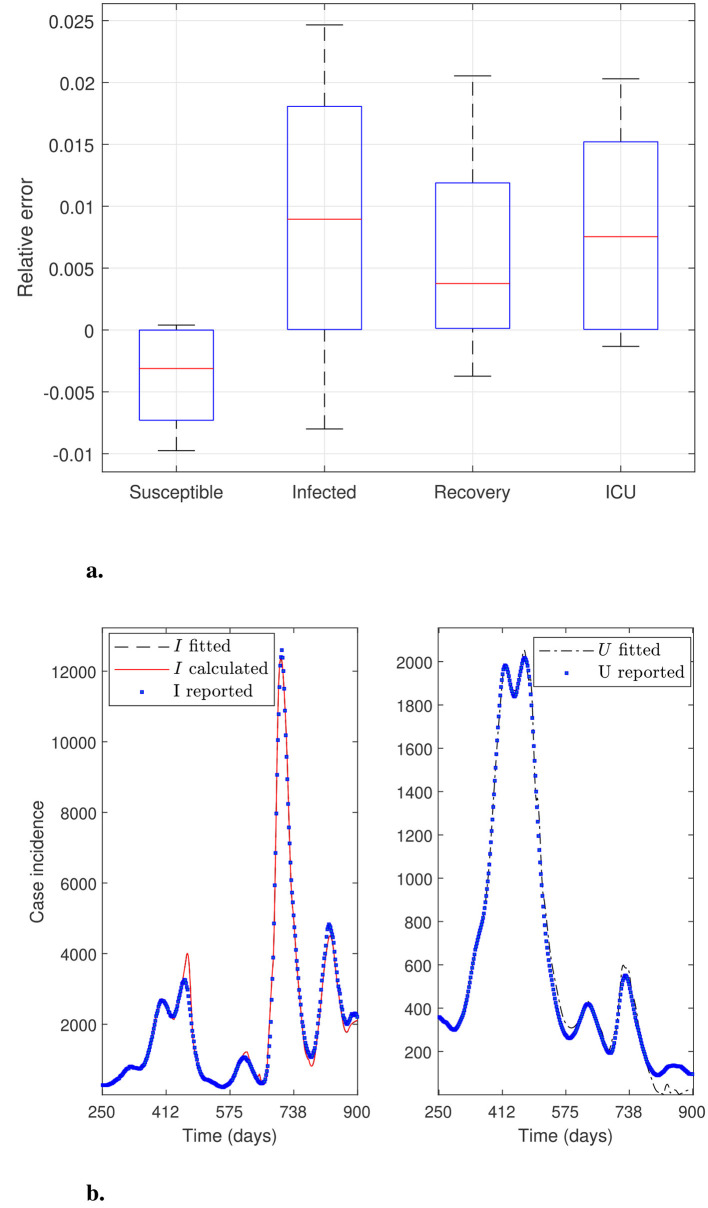
General modeling framework calibration using all modeling variables. Results correspond to the daily case incidence fitted by minimizing the objective function defined by Equation 5, adding the relative residuals for variables *R* and *S*. **(a)** depicts the relative error distribution of all modeling variables, and **(b)** incidence curves in the RM of Chile for variables *I*, *I*_*f*_, and *U*. Calibration is outstanding.

This section aims to solve modified parameter estimation problems with alternative objective functions to demonstrate whether the general modeling framework can effectively adjust the curve for variable *U*. This is relevant because the trend in ICU hospitalizations is the most appropriate indicator of an infectious disease's impact on the public health system. In addition, evaluating the general modeling framework's adaptability is relevant when considering the inclusion of other modeling variables, provided associated data are available. We obtain alternative objective functions by adding or dropping modeling variables and their corresponding data to the sum of squares defined in Equation 5. Furthermore, we use *N*_θ_0__ = 40 time subintervals to reconstruct the time-varying parameters and assess whether fitting accuracy increases (*N*_θ_ = 249 parameters to estimate). Figures 3–6 illustrate the effectiveness of the general modeling framework in achieving accurate calibration using only variable *U* up to the four variables *S*, *I*_*f*_, *R*, and *U*, respectively. For instance, the results plotted in Figure 3 consider only the residuals of variable *U* in the sum of squares in Equation 5. Figure 4 considers *I*_*f*_ and *U*, and so on.

From Figure 6, we observe that using the four modeling variables, we achieve the best calibration (Figure 6b), and a median close to 0 and narrow boxes for relative error distributions (Figure 6a).

### 4.3 General modeling framework robustness

**Figure 7:**
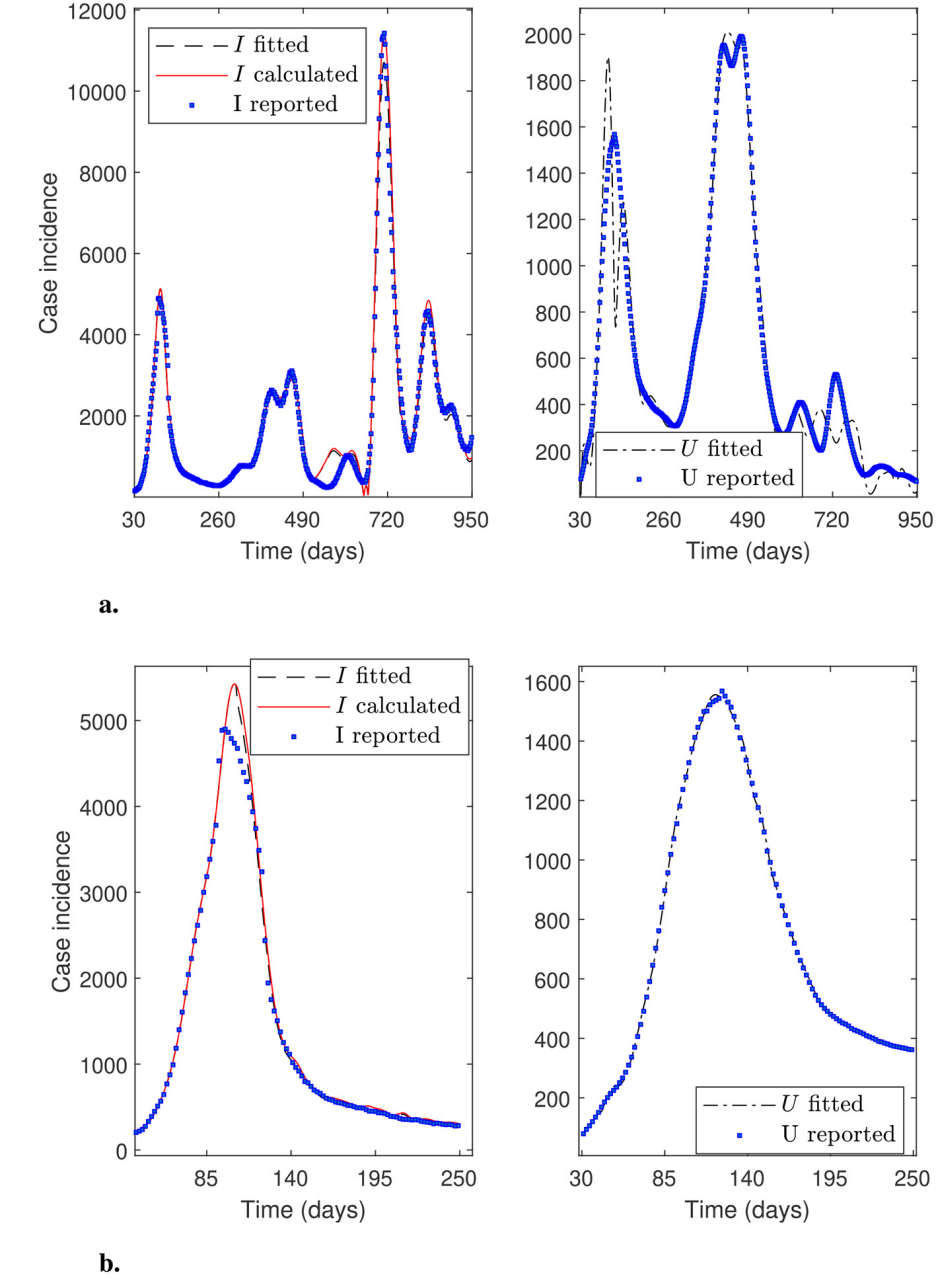
Fitted incidence curves for the Metropolitan región (RM, Central Chile in Figure 1). Results correspond to the daily case incidence. **(a)** shows a relatively good daily case incidence, calibrated for the almost entire pandemic, even though *I*_*f*_ and *I* peak spuriously near day 600. The fitted ICU-stay shows oscillations around the peak in the first wave and throughout the third wave. **(b)** shows an outstanding daily case incidence calibrated for the first wave.

**Figure 8:**
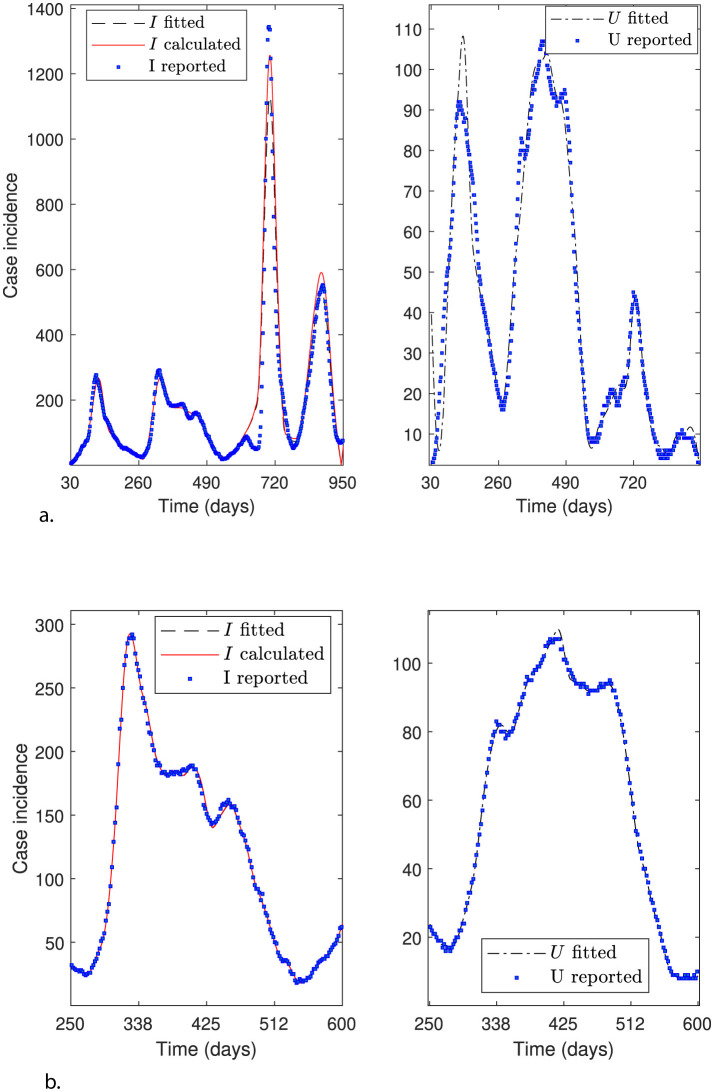
Fitted incidence curves for the Antofagasta region (Northern Chile in Figure 1). Results correspond to the daily case incidence. **(a)** shows a relatively good daily case incidence calibrated for the almost entire pandemic, even though variables *I* and *I*_*f*_ peak higher near day 600. The fitted ICU-stay shows oscillations near the end of the third wave. **(b)** depicts an excellent daily case incidence calibrated for the second wave.

**Figure 9:**
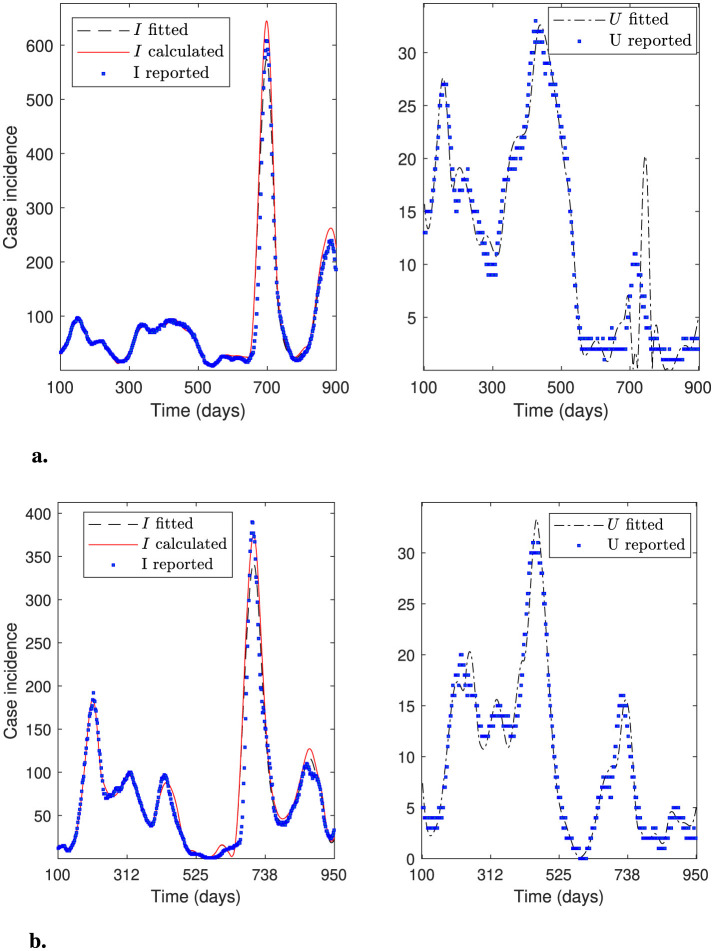
Fitted incidence curves for the Arica and Magallanes regions (far north and south of Chile in Figure 1). Results correspond to the daily case incidence. **(a)** depicts daily incidence curves calculated and fitted for the Arica and Parinacota region, and **(b)** for the Magallanes and the Antártica region for the almost entire pandemic. The two calibrations are relatively good, given the challenge of fitting the low daily case incidence reported in these remote, sparsely populated regions of Chile.

This section presents numerical experiments that demonstrate the robustness of the general modeling framework. When we select initial parameters using the general percentage decrease technique, the optimization solver consistently calibrates all datasets tested in this research. We obtained all the calibration results considering *N*_θ_0__ = 20 subintervals to reconstruct the time-varying parameters (*N*_θ_ = 129 parameters) and 30-day moving average to smooth the datasets. Table 3 presents quantitative metrics to verify the robustness of the general modeling framework applied to datasets from all of Chile, as explained in Sections 3.1 and 3.3, respectively. In addition, Figures 7–9 plot the case incidence fitted *I*_*f*_, calculated *I*, and reported *I*_*r*_ (denoted as “*I* fitted”, “*I* calculated”, and “*I* reported” in legends at left panels of figures). In addition, they plot the patients in the ICU, plus those dead confirmed by COVID-19, fitted *U* and reported *U*_*r*_ (denoted as “*U* fitted” and “*U* reported” in the legends of the right panels of the figures).

**Table 3:** General modeling framework robustness results.

**Region (figure)**	** $SS\left(\hat{\theta }\right)$ **	** $RMSE_{I_{f}}\left(\hat{\theta }\right)$ **	** $RMSE_{U}\left(\hat{\theta }\right)$ **	** $\hat{\delta }$ **	** $MRE_{I_{f}}\left(\hat{\theta }\right)$ **	** $MRE_{U}\left(\hat{\theta }\right)$ **	**Period**	**Loc**
Arica (Figure 9a)	2.51*e*−02	3.01*e*−03	2.83*e*−03	1.32*e*−01	4.20*e*−01	2.68*e*+00	100–900	North
Tarapacá	7.18*e*−02	4.62*e*−03	5.24*e*−03	8.12*e*−04	1.06*e*+00	5.34*e*−01	100–900	North
Antofagasta (Figure 8b)	8.75*e*−05	3.09*e*−04	2.39*e*−04	3.47*e*−05	2.73*e*−01	2.40*e*−02	250–600	North
Antofagasta (Figure 8a)	5.83*e*−01	1.52*e*−02	1.05*e*−02	2.76*e*−01	5.56*e*−01	3.24*e*−01	30–950	North
Atacama	8.11*e*−01	1.30*e*−02	1.95*e*−02	8.27*e*−04	8.95*e*−01	3.31*e*−01	100–900	North
Coquimbo	1.57*e*−02	1.99*e*−03	2.59*e*−03	9.77*e*−03	1.01*e*+00	2.60*e*−01	100–900	North
Valparaiso	1.31*e*−02	2.54*e*−03	1.96*e*−03	2.74*e*−03	4.18*e*−01	1.12*e*+00	250–950	Center
Metropolitan (Figure 7a)	2.47*e*+00	1.43*e*−02	3.52*e*−02	3.14*e*−05	4.58*e*−01	1.35*e*+00	30–950	Center
Metropolitan (Figure 7b)	2.30*e*−03	1.61*e*−03	2.18*e*−03	5.06*e*−03	3.75*e*−01	1.02*e*−02	30–250	Center
O'Higgins	8.37*e*−01	5.71*e*−03	2.24*e*−02	9.05*e*−04	7.44*e*−01	3.15*e*−01	100–900	Center
Maule	2.86*e*−01	7.56*e*−03	1.17*e*−02	3.67*e*−03	4.47*e*−01	6.70*e*−01	100–900	Center
Ñuble	2.86*e*−02	3.24*e*−03	2.98*e*−03	1.88*e*−06	4.97*e*−01	2.92*e*−01	100–900	Center
Biobío	1.76*e*−01	3.83*e*−03	9.84*e*−03	2.97*e*−07	8.37*e*−01	2.79*e*−01	100–900	South
Araucania	7.47*e*−02	4.96*e*−03	4.78*e*−03	6.68*e*−06	4.91*e*−01	8.38*e*−01	100–950	South
Los Ríos	6.52*e*−02	4.53*e*−03	4.58*e*−03	3.84*e*−06	1.54*e*+00	3.50*e*−01	100–900	South
Los Lagos	2.81*e*−02	1.86*e*−03	3.94*e*−03	2.55*e*−07	1.13*e*+00	1.28*e*−01	100–900	South
Aysén	1.62*e*−01	5.12*e*−03	9.16*e*−03	1.56*e*−06	1.26*e*+00	2.72*e*−01	100–900	South
Magallanes (Figure 9b)	2.28*e*−02	2.52*e*−03	2.85*e*−03	8.28*e*−02	4.56*e*−01	2.26*e*−01	100–950	South

**Figure 10:**
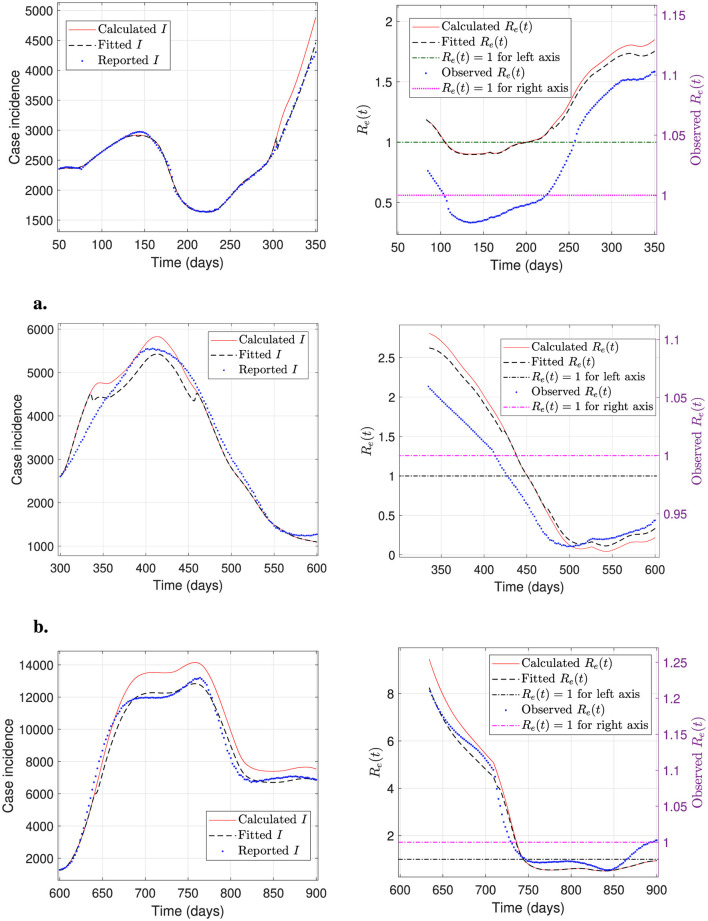
Incidence curves and effective reproductive number at a national level. The plots depict daily case incidence calculated, fitted, and reported, and the corresponding effective reproductive number *R*_*e*_(*t*), calculated, fitted, and observed over different periods/waves of the pandemic. The results show excellent agreement between the incidence curves and *R*_*e*_(*t*), and between the calculated and observed *R*_*e*_(*t*). **(a)** Period 50–350 (first wave). **(b)** Period 300–600 (second wave). **(c)** Period 600–900 (third wave).

In addition to the calibration performance demonstrated by our general modeling framework, we estimate the effective reproduction number from the national incidence curve. We show the results in Figure 10 for different periods/waves of the pandemic.

## 5 Discussion

This section analyses the results obtained in the preceding section. First, we demonstrate that by employing the general percentage decrease technique to select initial parameters, the *dde23* MATLAB solver enables stable evaluation of general epidemiological modeling. Second, we analyze alternative objective functions for parameter estimation, including modeling variables *S* and *R* (in addition to *I* and *U*), to demonstrate the adaptability of our framework. In fact, the general modeling framework is adaptable, as it can calibrate part or all of the model variables using partial or complete datasets collected in (11). Finally, we verify the robustness of the general modeling framework by demonstrating that it can calibrate any dataset under general conditions, independent of the data, as outlined in Algorithm 1.

### 5.1 General modeling framework is numerically stable

Table 2 shows that the calculation stabilizes after the fifth iteration of Algorithm 1, in which it exceeds the computational capacity and incurs the highest computational cost, leading to overflow. In addition, in iterations 6 and 7, it reaches stability and the stopping criteria (i) and (ii) in Equations 6a and 6b, respectively. In effect, it returns initial parameter values so that the corresponding computed curve *I*_*f*_(*t*) satisfies the devised general conditions.

Figure 2b (I5) illustrates “highly oscillating” curves for variables *I*_*f*_, *I*, and *U*, where variable *U* has the highest mean relative error (Table 2, Iter 5), and whose distribution has the most prolonged whisker, numerous outliers, and a long box. Conversely, in I6 and I7, the mean relative error for *U* is the smallest (marked in bold in Table 2), and the corresponding computed curves are significantly more stable than in I5. Note that Figure 2b in I7 depicts the initial curves for variables *I*_*f*_, *I*, and *U* used in the optimization solver's first iteration.

In summary, the general percentage decrease technique provides stability to the general modeling framework, ensuring that NaNs are no longer present in calculations during iterations performed by the objective function evaluation and the optimization solver.

### 5.2 General modeling framework is adaptable

Figure 3 shows excellent calibration of the general modeling framework for variable *U* using only *U*_*r*_ data, dropping the residuals for variable *I*_*f*_ from the sum of squares defined by Equation 5. The precision of this calibration is unmistakable in the corresponding relative error distribution depicted in Figure 3a, which shows a very narrow box, no whiskers, and a median close to zero. However, the framework does not fit variable *I*_*f*_ effectively without using the *I*_*r*_ data, as shown in Figure 3b. Relative error distribution for *I*_*f*_ in Figure 3a extends from −1 to 0.5 approx., underscoring the role of the *I*_*r*_ data besides the *U*_*r*_ data in the framework's robustness.

Figure 4 depicts calibration of the general modeling framework using variables *I*_*f*_ and *U* to minimize the original sum of squares in Equation 5. Figure 4b shows a good calibration, except for a spurious peak before day 575 for the fitted *I*_*f*_ curve. This anomaly would indicate poor counting for the incidence *I*_*r*_ of COVID-19 cases in the first wave, despite both variables' relative errors being very close to zero, as shown in Figure 4a. This discussion highlights the crucial need to count *I*_*r*_ data reliably for accurate parameter estimation.

Figure 5 depicts calibration of the general modeling framework using variables *I*_*f*_, *R*, and *U*. It considers the relative residuals already included for variables *I*_*f*_ and *U*, and adds those for variable *R* to the sum of squares given in Equation 5. Figure 5b shows a better calibration than Figure 4b for variables *I*_*f*_ and *U*. At the same time, their corresponding relative error distributions exhibit narrow boxes around a median close to 0 (relative error's median less than 1% for both variables and overall a relative error less than 2%), as depicted in Figure 5a. This finding suggests that fitting variable *R* may be suitable if the corresponding data were available and accurately counted by the health authorities, which was not the case during the first three months of the COVID-19 pandemic in Chile. It is important to note that the least narrow box is for variable *R*, although it was not counted during the first three months of the pandemic. It is worth noting that as we add more descriptive variables to the objective function and increase the number of subintervals (*N*_θ_0__ = 40), the overfitting becomes more evident (Figure 6b). However, given the real-world scenario, where we have data on infection incidence and ICU admissions, our framework can accurately fit both variables adaptively using the available data.

In summary, our general modeling framework can accurately and adaptively fit the peaks and trends of observed curves, which is particularly relevant for monitoring and informed government decision-making during health emergencies caused by transmissible diseases. In addition, our results suggest that it is crucial to include the reported incidence curve with a reliable count to achieve the highest accuracy. Regarding the addition of other modeling variables, such as *S* and *R*, we highlight that even if the corresponding data were unavailable, it would still be possible to use them to anticipate epidemiological scenarios, like those of the COVID-19 pandemic, by using synthetic data to inform the general modeling framework (4, 16).

### 5.3 General modeling framework is robust

From Table 3, we observe more appreciable step sizes $\hat{\delta }$ for Antofagasta, Arica, and Magallanes datasets, which correspond to the regions of Chile with the fewest populations. In this regard, the approximate solution $\hat{\theta }$ obtained by our general modeling framework is not necessarily the “best optimal solution”. In theory, the optimization solver could eventually iterate more times to obtain a better solution, thereby decreasing the step size. However, the relevance of our framework lies in the fact that it calibrates the observed curves and their general trends, as we demonstrated in Table 3 and Figures 7–9, which could contribute to anticipating an epidemiological outbreak and aiding policymakers in public health decision-making.

A general observation from Table 3, is that ${\text{MRE}}_{X}\left(\hat{\theta }\right)$ is greater than the ${\text{RMSE}}_{X}\left(\hat{\theta }\right)$ for both calibrated variables *X* = *I*_*f*_ and *X* = *U*. This finding aligns with the fact that ${\text{RMSE}}_{X}\left(\hat{\theta }\right)$ is computed with the residuals relative to the model variables, whose sum of squares we minimized (see Equation 5), whereas ${\text{MRE}}_{X}\left(\hat{\theta }\right)$ considers the residuals relative to the observed variables. In addition, this finding aligns with the differences between the calibrated and observed corresponding curves in terms of peaks and eventual numerical oscillations, as shown in Figures 7–9, which we discuss in detail below.

Figure 7a shows an overall good calibration for variable *I*_*f*_, whereas it is moderately good for variable *U*. The ${\text{RMSE}}_{X}\left(\hat{\theta }\right)$ and ${\text{MRE}}_{X}\left(\hat{\theta }\right)$ for variables *X* = *I*_*f*_ and *X* = *U* in Table 3 corroborate this finding (Metropolitan, period 100–900). Figure 7b shows an outstanding calibration for variables *I*_*f*_ and *U*, even though there was poor data counting in the first wave. By “poor data counting”, we mean the fact that the infected were consistently underestimated, particularly during the first wave, when the health system was not well prepared to conduct accurate follow-up; see (4, section 2.2.4). The ${\text{RMSE}}_{X}\left(\hat{\theta }\right)$ and ${\text{MRE}}_{X}\left(\hat{\theta }\right)$ for variables *X* = *I*_*f*_ and *X* = *U* in Table 3 (Metropolitan, period 30-250) corroborate this finding. In addition, the and ${\text{MRE}}_{U}\left(\hat{\theta }\right)$ is only around 1%, which agrees with the almost perfect calibration for *U*, as shown in Figure 7b. In the first case (Figure 7a), the oscillations in the fitted curve for *U* cause a higher ${\text{MRE}}_{U}\left(\hat{\theta }\right)$.

Figure 8b shows a good calibration for variable *I*_*f*_ and outstanding for variable *U*. The ${\text{RMSE}}_{X}\left(\hat{\theta }\right)$ and ${\text{MRE}}_{X}\left(\hat{\theta }\right)$ for variables *X* = *I*_*f*_ and *X* = *U* in Table 3 corroborate this finding (Antofagasta, period 250-600). The ${\text{MRE}}_{U}\left(\hat{\theta }\right)$ is the least, which agrees with the almost perfect fitting for variable *U*. Figure 8a shows a good calibration for variables *I*_*f*_ and *U*, including the first wave in the Antofagasta Region. The ${\text{RMSE}}_{X}\left(\hat{\theta }\right)$ and ${\text{MRE}}_{X}\left(\hat{\theta }\right)$ for variables *X* = *I*_*f*_ and *X* = *U* in Table 3 corroborate this finding (Antofagasta, period 100-900). However, the ${\text{MRE}}_{X}\left(\hat{\theta }\right)$ is higher for both variables *X* = *I*_*f*_ and *X* = *U* for Antofagasta Region in the period 30–950 than in the period 250-600, which excludes the first wave. This agrees with a higher peak for *I*_*f*_ near day 600 and *U* near day 700. By excluding the first wave (Figure 8b), the result is better due to the poor data counting during that period, as mentioned before.

Figure 9 shows results from regions in the far north (Figure 9a) and far south (Figure 9b) of Chile, where the distribution of *U* is very similar despite their geographical distance. The far north of Chile (Arica and Parinacota Region) exhibits the highest average relative error ${\text{MRE}}_{U}\left(\hat{\theta }\right)$ due to the low number of ICU admissions compared to the reported incidence. In some cases, only one patient per day was admitted to the ICU, whereas the model's estimate was three, implying a relative error of up to 200%. In contrast, Figure 9b  shows an outstanding calibration for variables *I*_*f*_ and *U* in the Magallanes and Antártica Region. ${\text{RMSE}}_{X}\left(\hat{\theta }\right)$ and ${\text{MRE}}_{X}\left(\hat{\theta }\right)$ for variables *X* = *I*_*f*_ and *X* = *U* in Table 3 corroborate this finding (Magallanes, period 100–950). The fitting is better for the variable *U*, which aligns with a lesser ${\text{MRE}}_{U}\left(\hat{\theta }\right)$. Remarkably, the calibration of the Arica and Magallanes dataset presented an additional challenge due to the small magnitude of the data and the smallest population size.

Finally, from Figures 10a–c, we observe an excellent agreement between the observed and calculated effective reproductive numbers (right panels). In addition, these numbers are in accordance with the respective incidence curves (left panels), i.e., when the incidence increases (resp., decreases) *R*_*e*_(*t*)>1 (resp., *R*_*e*_(*t*) < 1).

In summary, our general modeling framework captures trends observed in epidemiological curves across all regions of Chile, with varying population sizes and distinct periods/waves of the COVID-19 pandemic, including the effective reproductive number at the national level, further validating the results. Therefore, it can robustly optimize parameters to assess any COVID-19 dataset under general conditions, as determined by the initial parameter selection using the general percentage decrease technique and our careful implementation of the optimization solver.

## 6 Conclusions

Our research provides a robust modeling framework that accurately calibrates epidemiological curves under general conditions, independent of the target dataset. Also, the framework's calibration is adaptable to available data and reproducible using the code and datasets available in (11).

In the future, we plan to address the following research. First, we must expand the predictive capabilities of our framework. In this sense, in our previous work (4), we successfully predicted trends for the observed COVID-19 curves in the Metropolitan region of Chile using a parametric bootstrapping technique, as devised by (17). Second, a natural step is to apply our framework to other transmissible diseases, thereby further expanding its applicability. Third, another natural step is to compare our framework with other modeling approaches, using our unique overall numerical algorithm. Fourth, we would like to investigate the stability and calibration capabilities of our framework, which poses a challenge to investigating the model's output stability as a function of parameters. This last is a relevant aspect to achieve robust parameter optimization that remains open in general. In this sense, we aim to generate synthetic epidemiological curves using artificial neural networks that map all COVID-19 datasets in Chile, thereby strengthening parameter optimization (16). We can also apply synthetic data generation to expand the framework's predictive capabilities, rather than using a parametric bootstrapping technique, which has a high computational cost (4). Finally, we would like to test a hybrid optimization methodology to address parameter identifiability, as proposed by (18), which combines simulated annealing with nonlinear least-squares techniques.

In conclusion, our research represents a significant first step in establishing a robust modeling framework to investigate the dynamics of any transmissible disease. Our findings not only provide a solid foundation for future studies but also have the potential to inform the development of effective disease control strategies, thereby advancing public health.

## Data Availability

Publicly available datasets were analyzed in this study. This data can be found here: https://datos.usach.cl/dataset.xhtml?persistentId=doi:10.60547/USACH/44EVS0.

## Footnotes

1 In parallel computing is the delay between a request and its response caused by communication between processors and memory.
